# Compressional wave velocity measurements on mafic–ultramafic rocks under high aqueous fluid pressure and temperature help to explain low-velocity zones in the lithosphere

**DOI:** 10.1038/s41598-021-92248-2

**Published:** 2021-06-28

**Authors:** Evgeny B. Lebedev, Hartmut Kern, Ninely I. Pavlenkova, Oleg A. Lukanin, Konstantin V. Lobanov, Andrey V. Zharikov, Till Popp

**Affiliations:** 1 Vemadsky Institute of Geochemistry and Analytical Chemistry of the Russian Academy of Sciences, Kosygina Str. 19, Moscow, Russia 119991; 2 Institut Fur Geowissenschaften der Universitat Kiel, Olshausenstrasse 40-60, 24098 Kiel, Germany; 3 Schmidt Institute of Physics of the Earth of the Russian Academy of Sciences, B. Gruzinskaya Str. 10, Moscow, Russia 123242; 4 Institute of Geology of Ore Deposits, Petrography, Mineralogy and Geochemistry of the Russian Academy of Sciences, Staromonetny per. 35, Moscow, Russia 119017; 5 Lnstitut Für Gebirgsmechanik, Friederikenstraße 60, 04279 Leipzig, Germany

**Keywords:** Geochemistry, Geophysics

## Abstract

Deep seismic studies have revealed that low-velocity zones mainly occurred in the continental lithosphere at the depth of 100–150 km. Their origin has not been clearly explained yet. The article demonstrates the possible scale of Vp changes in crystalline rocks of different composition. The conclusions were made on the basis of the comprehensive analysis of the experimental data obtained by the authors. The compressional wave velocities in the temperature range from 20 to 800 °C, both in dry conditions (at pressure of 600 MPa) and in the presence of aqueous fluid (at pressure of 300 MPa) were measured. It is shown that the most significant decrease of velocities (by ~ 3 km/s) in the temperature range of 400–700 °C, corresponding to the deep waveguides of the lithospheric mantle, occurs under water pressure in ultramafic rocks enriched by olivine (dunites). Such decrease is due to rock structure changes caused by olivine serpentinization reactions. It is assumed that serpentinization and/or formation of similar hydrous minerals, which are stable in a wide range of PT-conditions in olivine-rich mantle rocks due to the influence of deep fluids, may cause low-velocities zones in the upper mantle at depths of about 100 km.

## Introduction

Deep seismic studies were carried out in the last century using peaceful nuclear explosions. Among them the studies performed by the United States and Canada within the framework of the Project Early Rise^1,2^*.* Nuclear explosions were produced in the Lake Superior in the center of the continent and a series of diverging seismic profiles were recorded. In Russia (Soviet Union) a large system of so-called ultra-long seismic profiles using peaceful nuclear explosions was developed as well^3,4^. The studies gave the data on the structure of the entire upper mantle and the transition zone to the lower mantle to a depth of more than 700 km and have revealed a number of unusual structural elements. Their origin has not been clearly explained yet.

**Figure 1:**
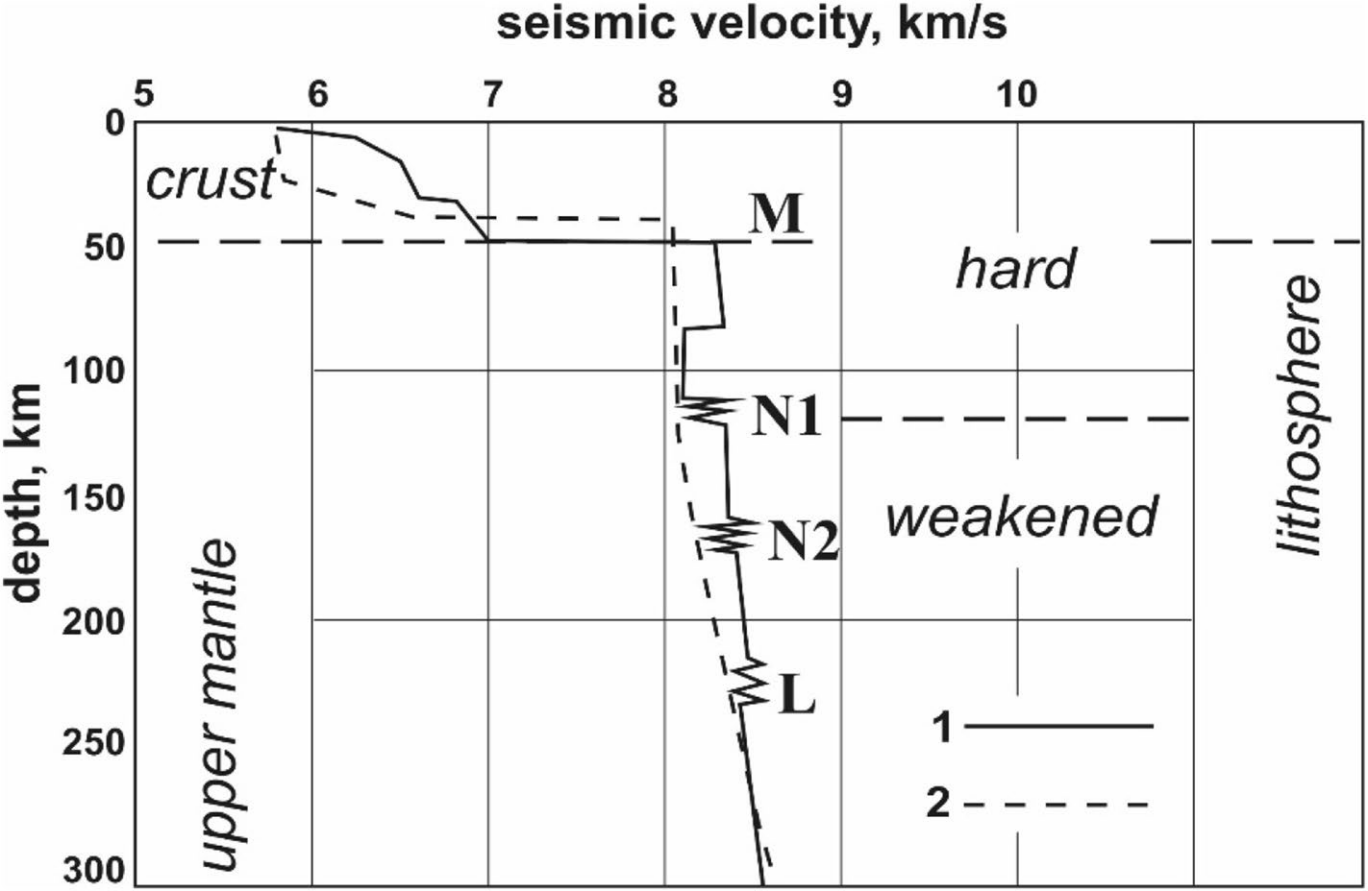
Generalized velocity model of the upper mantle of Northern Eurasia, built on a Super-long profile^6^ (curve 2) in comparison to a reference seismological model IASP-91 (curve 1). Seismic boundaries N1, N2, and L are shown how multi-layer tutu. M—sole of the Earth's crust.

An unexpected result of these studies was the discovery of the low velocity zones (layers) in the thick (250–300 km) craton lithosphere at the depth of 100–150 km^5,6^ (Fig. 1). These layers are underlain by a complex seismic boundary N (8° discontinuity in^7^), exposing thin layers of different velocities.

Low-velocity zones, also called waveguides, were discovered in the Earth's crust and upper mantle by seismologists at the beginning of the last century. The subhorizontal extended zones are characterized by a contrast decrease in seismic velocities, compared with the underlaying and overlaying rocks. So, if the source (earthquake) is located inside the zone or the incident wave falls at an angle greater than the angle of total internal reflection, then a channel wave is formed, propagating inside the layer, reflecting and refracting on its top and bottom, as in a waveguide.

In the past, low-velocity zones were distinguished in the upper mantle by seismological data (the Gutenberg model)^8^. They were explained by partial melting of mantle material (the asthenosphere), but this explanation is unlikely for the cratons lithosphere. Data on deep xenoliths and laboratory studies of physical properties of upper mantle rocks have shown that seismic velocities do not depend solely on the rock composition, but also on temperature and pressure. Within the ancient and young platforms of Northern Eurasia, the temperature derived from the seismic velocity at depths of 100–150 km doesn´t exceed 600–700 °C, which is not high enough for melting of upper mantle mafic–ultramafic rocks in dry conditions^9,10^.

Laboratory data on the physical properties of upper mantle rocks show that low-velocity layers and complex seismic boundaries in the upper mantle may be caused by sharp changes in the rock physical properties as a result of their structural and mineral phase transformations, controlled by effective pressure and temperature^11–16^, as well as by influx of deep fluid phases and their interaction with rocks^17–25^. So, we can assume that waveguides are presented by layers with high porosity and a high concentration of deep fluids. This explanation is confirmed by the laboratory studies data, on seismic velocities reduction in the fluid saturated materials^16,19,20,24,25^. This interpretation is also consistent with the electromagnetic studies results, showing that the N zones at a depth of 100–150 km often has a higher conductivity^26^.

## Methods of research

Here we present the laboratory data on compressional wave velocities (Vp) determined in some crustal and mantle rocks under high pressure and temperature under dry conditions and in the presence of aqueous fluids. The experiments under dry conditions were carried out in a triaxial multi-anvil high P/T apparatus under constant confining pressure of 600 MPa and at temperature up to 700°C^11–16^. The experiments with the presence of aqueous fluids were carried out in an apparatus with internal heating also under constant confining pressure of 300 MPa and at temperature up to 800 °C. The rock sample was not sealed. So, in this case the pore fluid pressure and confining pressure were in balance^19,20,23,24^. In contrast, in the experiments in dry conditions the effective pressure corresponds to the external loading and the fluid pressure is near-zero.

## Scale of elastic wave velocity changes at high PT-parameters at dry and wet conditions (experimental data)

**Figure 2:**
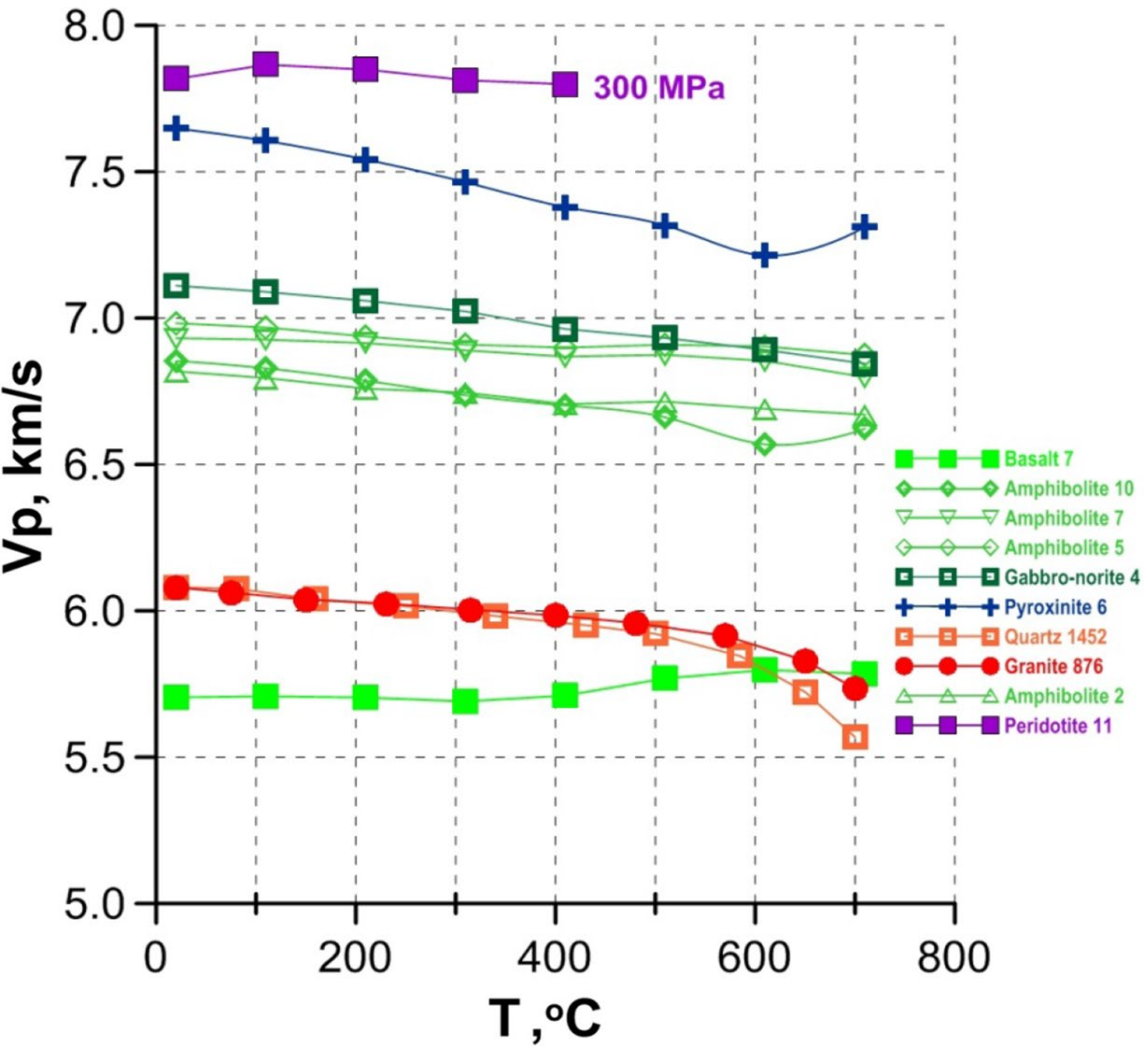
Vp in rocks under pressure of 600 MPa and at temperature up to 700 °C under dry conditions^11–14^.

**Figure 3:**
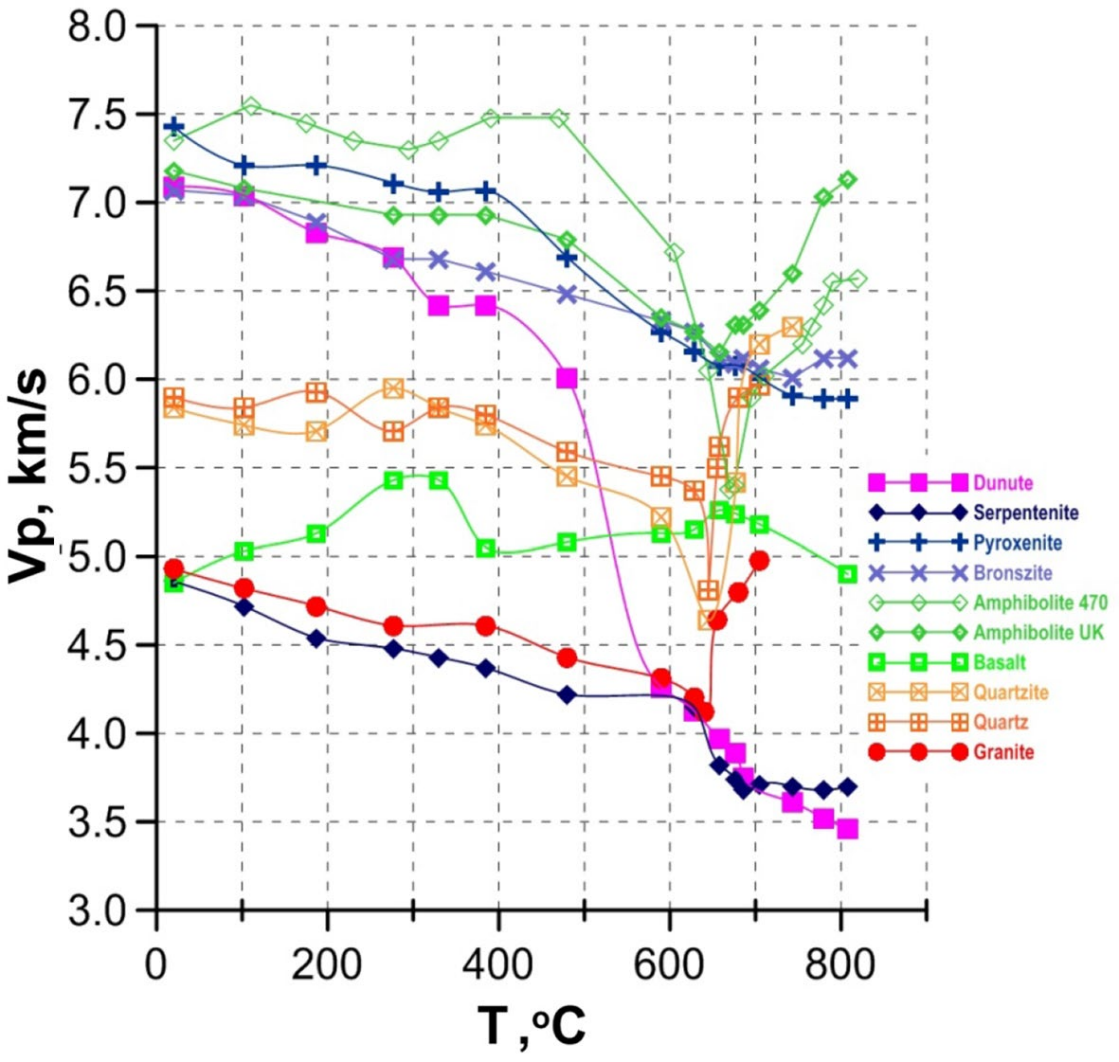
Vp in rocks under water pressure of 300 MPa and at temperature up to 800°C^16,19,20,24^.

The results of experimental determination of compressional wave velocities rates in rocks of mafic–ultramafic composition, which are supposed to form mainly the deep zones of the lithospheric upper mantle (peridotite, dunite, pyroxenite, serpentinite, amphibolite and basalt), are shown in Fig. 2 and 3. In addition, experimental data on acidic rocks (granite and quartzite, as well as pure quartz) are presented for comparison. Details on rock mineral and chemical composition, as well as the sampling sites, are given in^12–14,16,20,23,24^.

*In dry conditions*, the compressional wave velocities rates in the investigated mafic–ultramafic rocks change very slightly in the entire temperature range from 20 to 700 °C: with T increase the Vp value either remains almost constant, varying within ± 0.1 km/s (peridotite), or decreases slightly: not more than 0.3 km/s (pyroxenite, gabbro-norite, amphibolite) (Fig. 2). Rock composition effect is more significant. In the studied entire temperature range, Vp in the ultramafic rocks is 0.3–0.5 km/s higher than in the mafic rocks. At the same time, mafic rocks including water-bearing phases like amphiboles, have similar or lower Vp values. However, the Vp difference for gabbros and amphibolites of different composition at this temperature does not exceed 0.3 km/s. Amygdaloidal basalts have significantly lower Vp values (at ~ 1 km/s), due to their higher porosity, heterogeneous structure, the content of glass in addition to crystal mineral phases, as well as, the presence of secondary minerals in the amygdules, including water-containing ones (Fig. 2).

**Figure 4:**
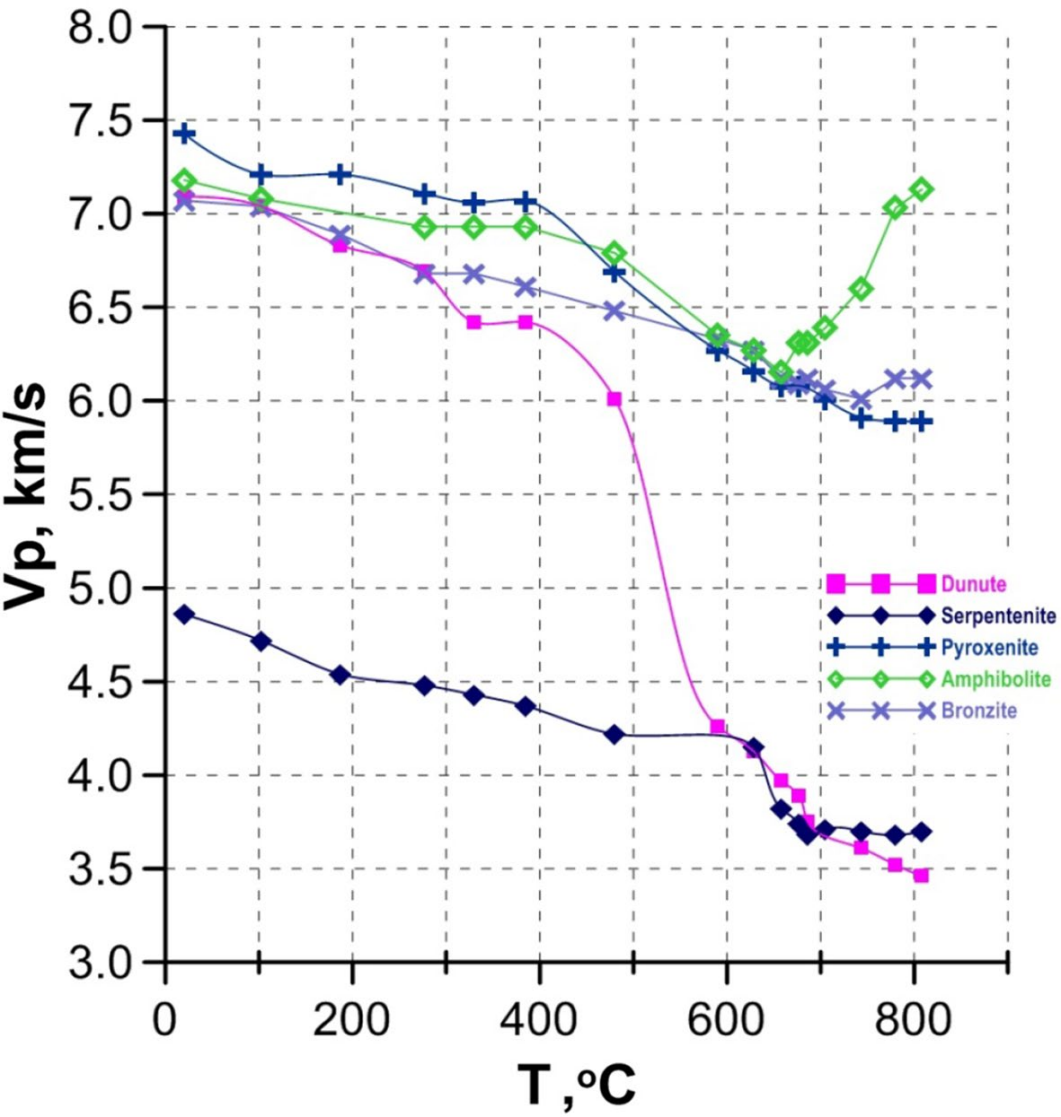
The Vp temperature dependence for mafic–ultramafic rocks under water pressure of 300 MPa.

*Under water pressure*, the Vp temperature dependence for mafic–ultramafic rocks is more complex in comparison with rocks measured under dry conditions and is mainly determined by the structural features and mineralogical composition of the samples (Fig. 3, 4). In dry conditions, Vp in pyroxenites decreases relatively monotonously with increasing temperature up to 800 °C. However, under water pressure the decrease in Vp is more pronounced by about 1 km/s, especially at temperatures above 400 °C. In basalt under water pressure in the range of 400–650 °C the elastic wave velocities vary relatively slightly, remaining at the level of 5–5.3 km/s. Temperature increase up to 800 °C leads to Vp decrease, which does not exceed more than 0.7 km/s.

The dependence Vp = *f (T)* for amphibolite and dunite demonstrates a completely different pattern. For amphibolite a relatively monotonous decrease of Vp value with temperature rise up to 500–600 °C is followed by an abrupt drop. So, the minimum Vp values are observed at temperatures of 650–670 °C. A further temperature increase leads to a sharp Vp increase. The value of the maximum velocity drop (ΔVp) in the range of 600–700 °C for amphibolites of different composition reaches 1.5–2 km/s.

A decisive role in the sharp drop in the elastic wave velocity in amphibolites can be played by microcracks opening due to thermal dilatancy caused by differences in thermal expansion of rock forming minerals enforced by water penetration into intergranular pore space^24^.The subsequent velocity increase in amphibolite at T > 670 °C is apparently caused by high-density water fluid penetration into the rock pore space and formation of an intergranular silicate water-enriched liquid as a result of partial melting. In addition, the presence of a small amount of quartz in the amphibolite may explain a minimum on the dependence Vp = *f(T)* by the alpha–beta phase transition.

The pronounced influence of the α-β phase transition in quartz on Vp with a minimum at 630–700 °C is observed in pure quartz (Fig. 3), in granite and quartzite, both under dry (Fig. 2) and wet conditions (Fig. 3).

A dramatic drop of Vp under water pressure is measured in all ultramafic rocks enriched by olivine^20^ (Fig. 4). In dunite under water pressure a gradual decrease in Vp is observed above about 150–200 °C followed by a sharp drop (ΔVp ≈ 3 km/s) in the range of 400–700 °C. At T ≈ 650–750 °C in dunite, Vp reaches the values that were measured under the same conditions in serpentinite. At low temperature, Vp in serpentinite is significantly lower (~ 2 km/s) than in dunite. Temperature increase up to 600 °C in serpentinite is accompanied by monotonous Vp decrease, but a further appreciable decrease of Vp by ~ 0.4 km/s in the relatively narrow range of 640–680 °C is observed. Increasing temperature up to 800 °C has practically no effect on compressional wave velocity.

**Figure 5:**
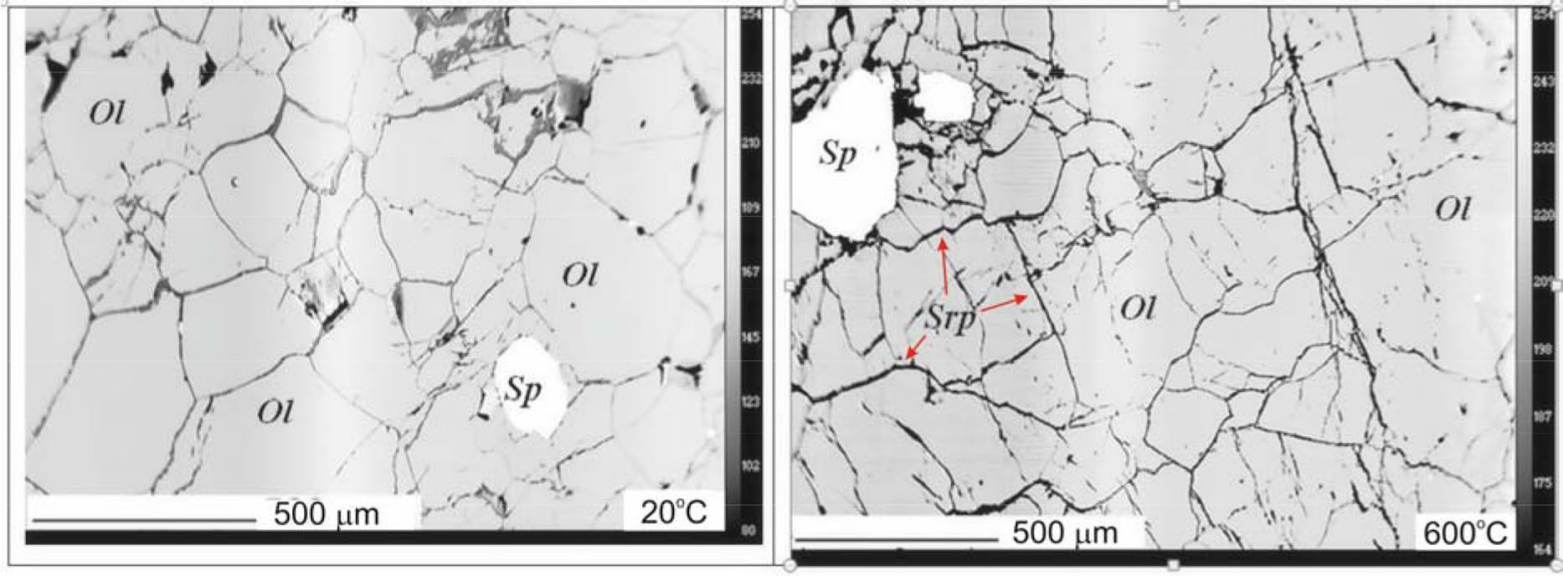
Changes in dunite structure with temperature increase under water pressure of 300 MPa (micrographs of the thin sections of quenched samples after experiments at 20 and 600 °C). Ol-olivine, Srp-serpentine, Sp-spinel.

At a first glance, dunite undergoes relatively insignificant structural transformation in the temperature range of the sharp decrease of Vp between 640–680 °C (Fig. 5). However, with increasing temperature the boundaries between olivine grains expand (“microcracks”), promoting fluid infiltration and formation of serpentine fillings, and at T > 600 °C, along with serpentine and another water containing mineral close to clinochlor is formed, containing about 12 wt.% H_2_O (Table 1). The newly formed serpentine minerals along grain boundaries gives rise for the observed sharp velocity decrease.

**Table 1:** Chemical composition of the initial samples studied in the experiments under water pressure and new formed minerals (wt.%)^16,20,23,24^.

Rocks, minerals	SiO_2_	TiO_2_	A1_2_O_3_	FeO	MnO	MgO	CaO	Na_2_O	К_2_O	P_2_O_5_	NiO	Cr_2_O_3_	Σ
Dunite	40.89	0.05	0.31	7.20	0.14	50.12	0.17	0.08	0.06	0.04		0.38	99.45
Serpentinite	37.96	0.01	0.03	4.13	0.04	39.86	0.03	0.02	0.01	0.03		0.05	82.18
Olivine*	40.49	0.01	0.02	6.78	0.15	52.07	0.10	0.01	0.01	0.06	0.37	0.04	100.1
Serpentine**	42.63	0.01	0.31	2.39	0.01	40.79	0.09	0.02	0.02	0.09	0.12	0.07	86.55
Clinochore**	30.13	0.03	17.74	1.97	0.02	34.72	0.05	0.56	0.02	0.10	0.10	2.20	87.64
Pyroxenite	52.88		1.96	6.21		16.99	21.02						99.06
Amphibolite 470	51.26	1.62	13.28	12.37	0.23	7.25	10.84	0.59	0.26	0.24			98.97
Amphibolite UK	49.59	1.79	12.85	15.10	0.27	5.77	10.82	1.82	0.25	0.17			98.96

*olivine in the original dunite; **minerals formed in dunite at P_H2O_ = 300 MPa and 600 °C. The concentration of H_2_O in serpentinite, serpentine and "clinochlore", approximating by the difference (100-Σ) is 17, 13 and 12 wt.%, respectively.

It should be noted that there is a strong decrease in Vp_,_ when serpentinization affects a very small volume of dunite (mainly along the boundaries of olivine grains), i.e. in order to cause this effect in olivine-containing rocks, a relatively small amount of water is sufficient, which is spent by the formation of water-containing minerals and filling the free space between the crystals.

Significantly less expressed in comparison with a sample of dunite the abrupt decrease in Vp in serpentinite at T > 640 °C is obviously due to phase reactions with the formation of talc^27^ and the resulting change in the internal structure of the sample.

## Discussion

**Figure 6:**
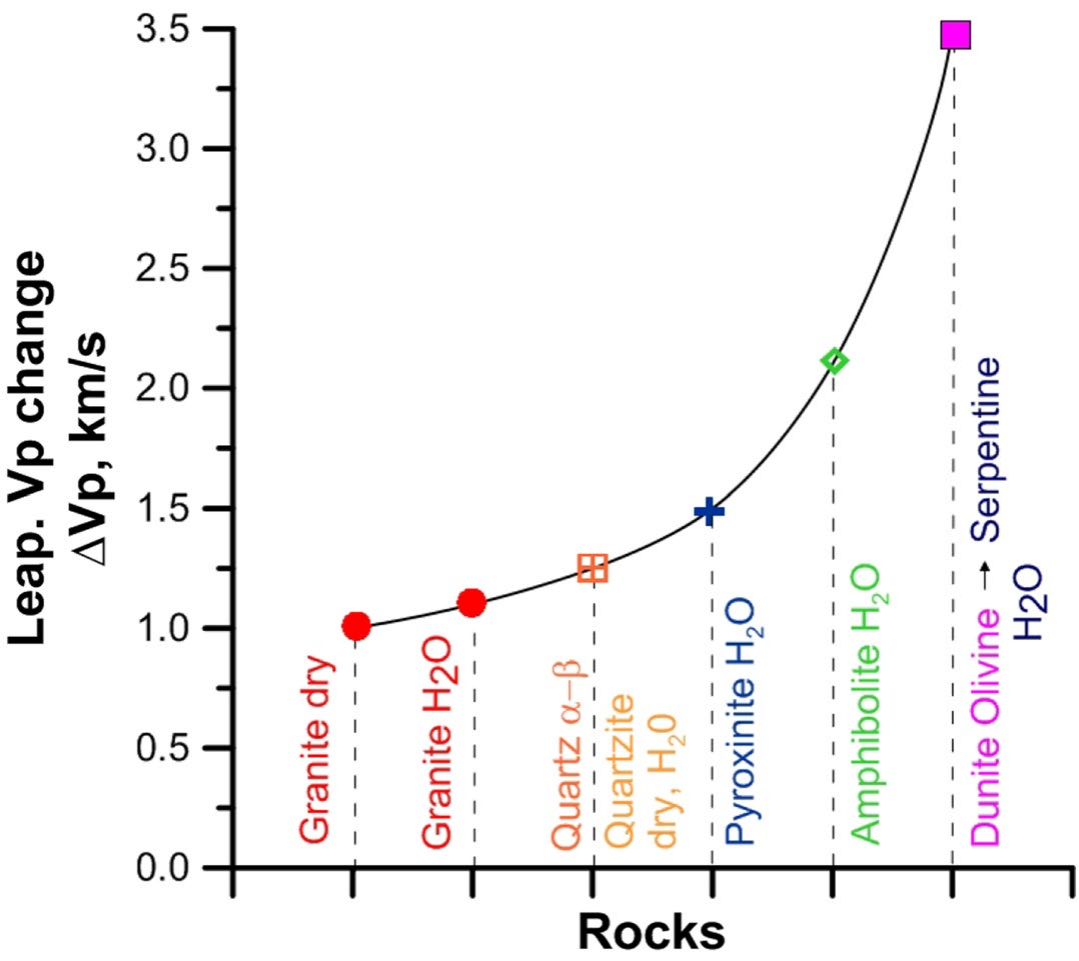
The value of ΔVp—changes of elastic wave velocities at temperatures of 400–800 °C. The figure shows experimental data in dry conditions^11–15^ and in the water presence^16,19,20,24^.

The scales of the possible drop of elastic wave velocities (ΔVp) in mafic–ultramafic and acidic rocks at high pressures in the temperature range of 600–700 °C, proper for deep waveguides in the lithospheric mantle, is shown in a generalized form in Fig. 6. The value of ΔVp for quartz-bearing rocks, due to the α-β transition in quartz and in dry as well as in wet conditions, can be 1–1.2 km/s. For pyroxenites, the drop in the elastic wave velocity is most clearly manifested under water pressure and is ~ 1.3 km/s. In addition to the rock textural and structural changes in the presence of water with temperature increase, it is likely due to partial amphibolization of pyroxenes at T > 400 °C. Obviously, this is why the ΔVp value for pyroxenites under water pressure is close to, but does not exceed the values found in amphibolites (≤ 1.5 km/s).

It should be noted that a more significant drop of the elastic wave velocity (up to 2 km/s) was observed in amphibolites. In this case, the effects of the quartz α-β transition and thermal dilatancy are obviously summed up.

The greatest drop of Vp in all mafic–ultramafic rocks under water pressure is found for dunite (ΔVp = 3–3.5 km/s). It is important that Vp at temperatures of 650–800 °C reaches values of 4–3.5 km/s, corresponding to the values typical for serpentinite. The exposure time in the experiments on Vp measurements in dunite within the entire temperature range was not enough for the completion of the reactions responsible for formation of equilibrium mineral associations, including serpentine and other water-containing phases. The serpentinzation processes may take place at lower temperatures (≤ 400 °C)). Hence, with a much longer exposure at such temperatures, a more significant decrease in Vp could be expected. At the same time, the experimental data obtained show that at the higher T (> 400–500 °C) in the water presence a sharp Vp decrease in dunite realizes in a very short time (hours). The effect is obviously caused by several factors: a change in the rock structure (microcracks opening as a result of aqueous fluid penetration into the rock), as well as formation of serpentine and other hydrous silicate water-containing phases along the grain boundaries of olivine. Thus, under PT-conditions of the upper mantle, the penetration of water fluid, even in relatively small amounts, can provoke a very sharp Vp drop in olivine-rich ultramafic rocks, which is much higher than in other ultramafic–mafic rocks (pyroxenites, amphibolites).

The experimental data of the present study show that serpentine and other water-containing high-pressure phases developing on olivine in the presence of water in the MgO-SiO_2_-H_2_O system can be stable up to 8 GPa (i.e., to depths of ~ 260 km) and 1100°C^28–31^. Under more higher pressure in conditions of the lower mantle and in the presence of water, wadsleite (a mineral modification of olivine), characterized by reduced elastic wave velocities compared to olivine, becomes the stable hydrated phase^32,33^. Thus, the effect of serpentinization or formation of similar water-containing minerals as a result of the interaction of water fluids with olivine can be applied to explain the nature of low velocities zones at much greater depths, characterized by the corresponding PT-conditions: about 100 km and below.

## Conclusion

The results of experimental studies of various rocks of a wide spectrum of compositions at high pressure in dry (600 MPa) and in water (300 MPa) conditions in the temperature range of 20–800 °C demonstrate strong water fluid effects on the elastic wave velocities caused by changes in the rock structure, as well as by mineral reactions and quartz alpha–beta phase transition.

It is found that a significant drop in the elastic wave velocity occurs under water pressure in the temperature range of 400–700 °C, corresponding to the conditions of deep waveguides in the lithospheric mantle, in rocks of mafic–ultramafic composition (dunites, pyroxenites, amphibolites).

The most dramatic velocity decrease (by 3 km/s) occurs in ultramafic rocks enriched by olivine (dunites) as a result of the changes in the rock structure and serpentinization reactions in olivine. For other rocks of both mafic–ultramafic and acidic composition, the Vp drop in this temperature range does not exceed 1.5–2 km/s.

The obtained data may be important for the nature of waveguides in the upper mantle determination. The influence of water as the main volatile component in deep processes at high pressures and high temperatures can lead to various magmatic and metamorphic reactions and changes in physical and chemical properties, which will need to be studied in the future.

The formation in olivine-rich mantle rocks under the influence of deep water fluids serpentine and/or other similar water-containing phases that are stable in a wide range of PT-conditions^28–33^ can be cause the low velocities zones occurrence in the upper mantle at depths of about 100 km and below.

## Supplementary Information

Supplementary Information.
