# Supplementary material for: Compressional wave velocity measurements on mafic–ultramafic rocks under high aqueous fluid pressure and temperature help to explain low-velocity zones in the lithosphere
Source: Sci Rep. 2021 Jun 28;11:13424. doi: 10.1038/s41598-021-92248-2 (PMC8239040; doi:10.1038/s41598-021-92248-2)
Supplement: Supplementary file 1 — Supplementary Information. [file 41598_2021_92248_MOESM1_ESM.pdf]

Additional information

Experimental setups and measurements techniques

Two setups were used for the experiments: a multi-anvil pressure apparatus (Fig. 1) and an internally heated gas apparatus (Fig.2). The principal differences between the two setups were as follows. The experiments with use of a multi-anvil pressure apparatus were carried out in dry conditions and under high effective pressure P_{eff}=P_{conf}=600 MPa. The experiments with use of internally heated gas apparatus were carried out with water fluid presence and, in contrast, under low effective pressure P_{pore}=P_{conf}=300 MPa.

Experiments in dry conditions. Multi-anvil apparatus

The setup was used for the experiments carried out in the ***Petrophysical lab of the Institut fur Geowissenschaften der Universitat Kiel, Germany.*** A state of near hydrostatic stress was achieved there by advancing six pyramidal pistons in three mutually orthogonal directions onto cube-shaped specimens (43 mm edge length). One end of each piston next to the specimen was surrounded by a furnace and heat is transmitted from the pistons to the specimen. Thus, a very homogeneous heating and distribution of temperature was obtained within the large-volume specimens. Temperature was measured using thermocouples placed in a cavity at the end of each piston very close (about 1 mm) to the specimen. The temperature drop between the thermocouples and the centre of the specimen was less than 5ºC at 700ºC as has been confirmed by temperature measurements at different places within a test sample. The ultrasonic transmission method was used for the elastic wave velocities measurements. 2 MHz and 1 MHz lead titanate zirconate (PTZ) transducers can generate both compressional and shear waves. The transducers were placed on the low temperature side of the pistons. The travel time of the pulses through the specimen was obtained by subtracting the calibrated time needed for the pulse to travel to and from the specimen through the pistons from the total time measured by the transducers. Length changes of the sample cubes due to changes of pressure and temperature are measured by the piston displacement. So, the calculated velocities refer to the actual dimensions of the cubes under PT-conditions. The accuracy of the velocity measurements was estimated to be better than 1%.

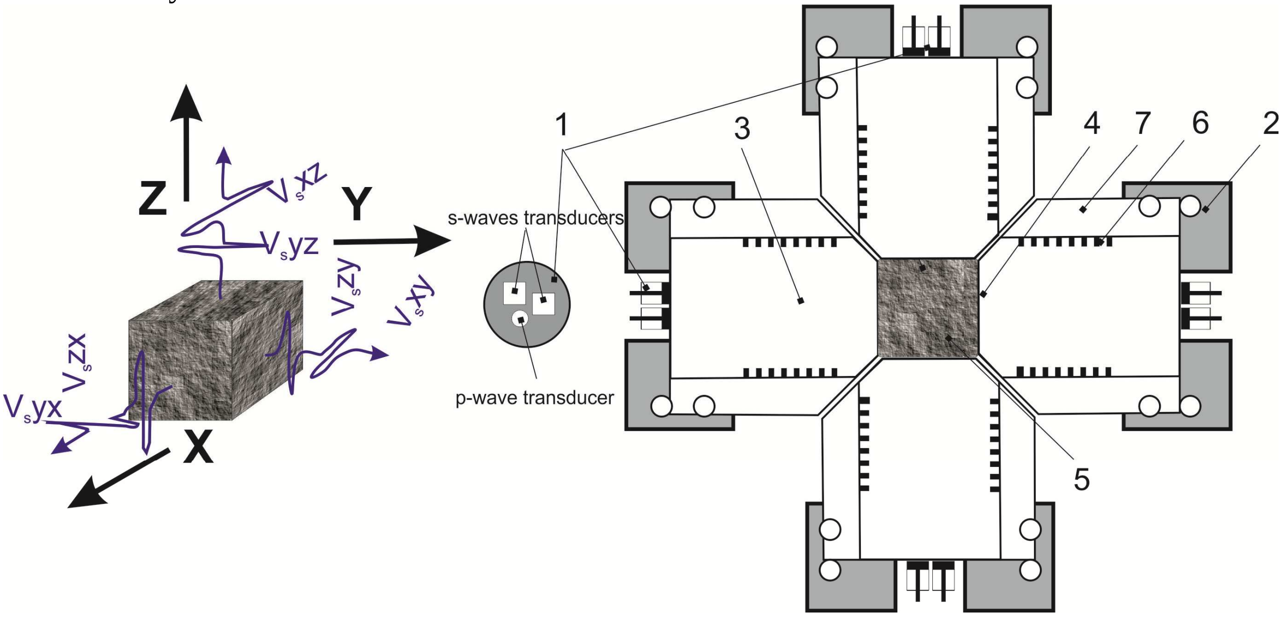

a **b**

Fig. 1. Schematics of the multi-anvil apparatus. (a) Sample reference system as related to inherent fabric elements (foliation XY-plane and lineation X-direction) of the sample cubes used in the multi-anvil apparatus. (b) Multi-anvil apparatus: 1 - transducer arrangement, 2 - cooling system, 3 - piston (buffer rod), 4 – thermocouple, 5 - sample, 6 - furnace, 7 - insulation.

The setup is capable to measure simultaneously the P- and S-wave velocities in the three orthogonal directions, first at increasing confining pressure (up to 600 MPa), and then at increasing temperature (up to 600ºC) at 600 MPa confining pressure. Elastic wave velocities were measured at intervals of about 50 MPa during pressure increase to 600 MPa. Maintaining the hydrostatic pressure constant, the temperature was increased in steps of about 80ºC over about 15-min periods. To ensure that the samples had reached pressure and temperature equilibrium, successive readings were taken at time intervals of at least 30 min.

Experiments under water presence. Internally heated gas apparatus

The aim of the experiments was to study the effect in the rock microstructure changes due to the simultaneous processes initiated by the presence of water fluids at high temperature on elastic and other physical properties. Therefore, the studies consisted of two parts: elastic wave velocities measurements at high PT and comparative studies of the rock composition, microstructure, and physical properties before and after high PT experiments on initial and quenched samples.

The experiments were carried out in the ***Vemadsky Institute of Geochemistry and Analytical Chemistry, Moscow, Russia*** using the gas apparatus with an internal heating system (Fig.2). The setup produced a true hydrostatic pressure up to 500 MPa with use of nitrogen as pressure-transmitting medium. A three-zone electric furnace was capable to heat the sample up to 1200 ^{o}C. The temperature was measured by three thermocouples.

Cylindrical not jacketed core samples of approximately 0.5 cm in length and approximately 0.8 cm in diameter were placed in a platinum reaction chamber that was then filled with water. So, a sample was in direct contact with fluid in the reaction chamber that ensured equality of the pore and confining pressures. The reaction chamber was closed by a mercury seal.

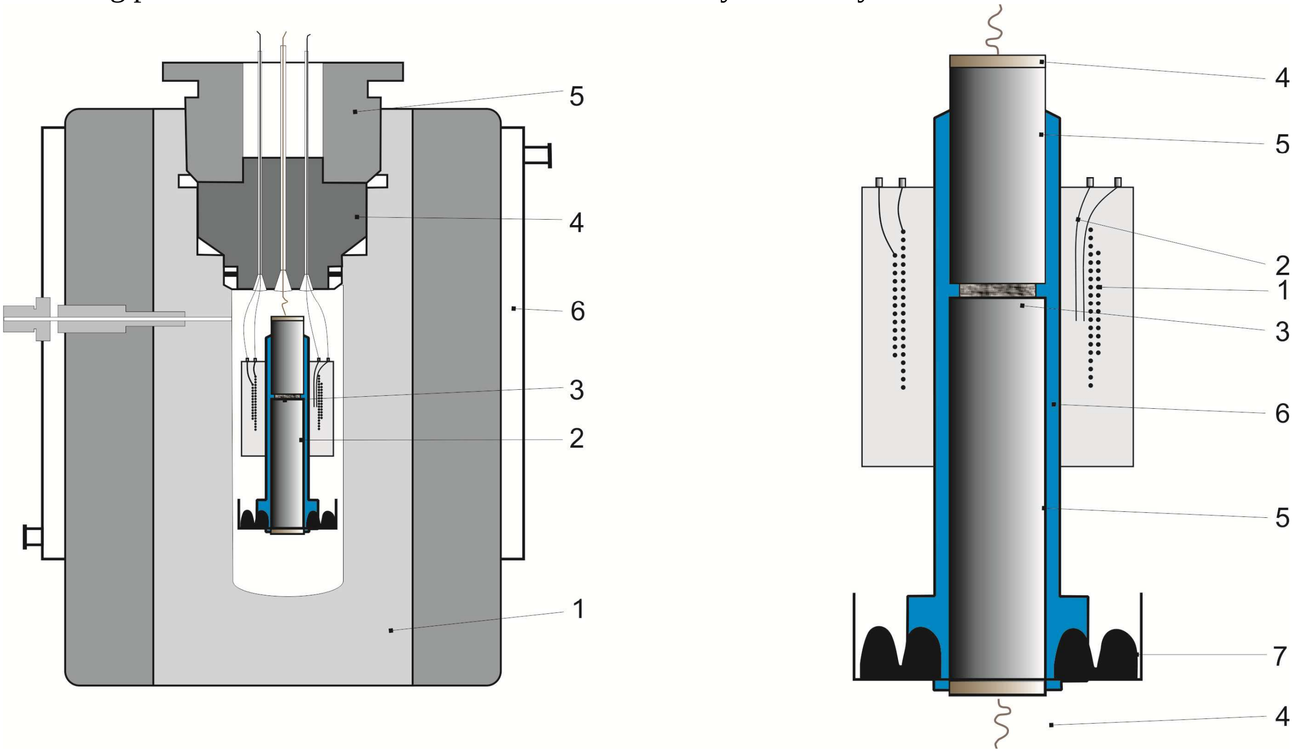

a b

Fig. 2. (a) Schematics of internally heated gas apparatus. 1 - pressure vessel, 2 - measuring cell, 3 - sample, 4 - head, 5 - closure nut, 6 - water cooler.
(b) Measuring cell of internally heated gas apparatus: 1 – furnace, 2 - thermocouple, 3 – sample, 4 - piezoelectric elements, 5 - acoustic buffer rod, 6 – container of water, 7 - mercury seal.

Piezoelectric P-wave (PZT ceramic transducers, 2 MHz frequency) were placed outside the heated area at the ends of two cylindrical stainless steel buffer rods. The compressional wave velocities in the rock samples were determined by ultrasonic pulse transmission in combination with the echo-impulse method. The acoustic wave generated by piezoelectric transducer passes through the buffer rod, then through the sample and the second buffer rod to the receiving transducer. The total travel time of P-wave was measured, the time for passing through the buffer rods obtained using echo-impulse method was subtracted from it, and, as a result, Vp in the sample was calculated. To ensure a confident separation of the transmitting and echo signals, the buffer rods of different lengths were used. The changes in the length of the sample due to thermal extension assumed to be negligible small (<1 %, Zaraisky & Balashov, 1995) and was taken into account in the calculation of the possible Vp measurement error.

The experiments on measuring the elastic wave velocities for the temperature range from 20 to 850°C lasted about 6–8 hours. The accuracy of the measurements was 5°C for temperature, 1% for pressure and 5% for velocity.

In order to study the changes in composition, structure and physical rock properties at high temperatures quenched samples were prepared. The samples were heated in water under pressure of 300 MPa to one of the given temperatures, kept for 3 or 24 h and was then cooled rapidly; the time of quenching was about 2-3 min.

Zaraisky G.P., Balashov V.N. Thermal decompaction of rocks. In: *Fluids in the crust. Equilibrium and transport properties.* (Shmulovich K.I., Yardley B.W.D., Gonchar G.G.), 253-284. (Chapman and Hall, 1995).

Table 1. The compressional wave velocity (Vp, km/s) in dunite, serpentinite, pyroxenite, amphibolite, basalt in the presence of water under high pressure of 300 MPa and temperatures (20-808°C) [16, 19, 20, 24].

**Temperature, °C**	20	102	187	277	330	385	480	590	628	658	677	686	705	743	780	808
Dunite	7.09	7.04	6.83	6.69	6.42	6.42	6.01	4.26	4.13	3.97	3.89	3.75	-	3.61	3.52	3.46
Serpentinite	4.86	4.72	4.54	4.48	4.43	4.37	4.22	-	4.15	3.82	3.74	3.68	3.71	3.7	3.68	3.70
Pyroxenite-Augite	7.43	7.21	7.21	7.11	7.06	6.07	6.69	8.27	6.16	6.08	6.08	-	6.01	5.94	5.91	5.91
Bronzitite	7.07	7.04	6.89	6.68	6.68	6.61	6.48	6.33	6.27	6.12	6.09	6.12	6.06	6.01	6.12	6.12
Amphibolite 470	7.45	7.64	-	-	7.33	7.48	-	6.60	5.82	5.69	**5.72**		5.91	5.94	6.15	6.45
Amphibolite UK	7.18	7.08	-	6.93	6.93	6.93	6.79	6.35	6.27	6.15	6.31	5.31	6.39	6.60	7.03	7.13
Basalt Kir	4.85	5.03	5.13	5.43	5.43	5.05	5.08	5.13	5.15	5.26	5.24	-	5.18	-	-	4.90

Table 2. The compressional wave velocity (Vp, km/s) in quartz, quartzite and granite in the presence of water under high pressure of 300 MPa and temperatures (20-700°C) [19,24].

**Temperature, °C**	20	102	187	277	330	385	480	590	628	640	645	655	658	680	705
Quartz (G.C.)	5.90	5.84	5.93	5.71	5.84	5.80	5.59	5.45	5.37		4.81	5.50	5.62	5.90	5.97
Quartzite (B.R)	5.84	5.74	5.71	5.95	5.84	5.74	5.45	5.22	5.03	5.41	-	-	-	-	6.30
Granite (El.)	4.93	4.82	4.72	4.61	-	4.61	4.43	4.31	4.20	4.12	-	4.64	-	4.80	4.98
